# Efficacy of extracts from *Datura Metel* L. for Psoriasis: a meta-analysis of case series and single-arm studies

**DOI:** 10.1186/s12906-023-04159-6

**Published:** 2023-09-14

**Authors:** Xiaopu Sang, Huanzhou Bi, Xinlei Si, Yihang Wang, Xianjie Shi, Fenfang Wu

**Affiliations:** Longgang Key Laboratory of Chinese Medicine and Immunology, Shenzhen Hospital, Beijing University of Chinese Medicine, Shenzhen, Guangdong, 518100 China

**Keywords:** Psoriasis, Meta-analysis, *Datura Metel*, Clinical studies, Treatment outcome

## Abstract

**Background:**

*Datura Metel* L. has been used to treat psoriasis in China for a long time. The effect of extracts from *Datura Metel* L. for Psoriasis has not been previously confirmed. This study aimed to evaluate the efficacy of extracts from *Datura Metel* L. for patients with psoriasis.

**Methods:**

PubMed, Cochrane Library, Embase, and other databases were searched from database inception until to September 1, 2021. A quality assessment and data extraction were performed by 2 independent reviews. We used a random-effects meta-analysis model to estimate the pooled curative effect, pooled odds ratio (OR), and 95% confidence intervals.

**Results:**

Nine studies were included in Meta-analysis, including a total number of 1778 patients with psoriasis. The case cure rate of *Datura Metel* L. intravenous therapy was 0.48 (95% CI: 0.33, 0.62) and of *Datura Metel* L. oral therapy was 0.42 (95% CI: 0.17, 0.68), respectively. The case effective rate of *Datura Metel* L. intravenous therapy was 0.91 (95% CI: 0.84, 0.97) and of *Datura Metel* L. oral therapy was 0.94 (95% CI: 0.88, 0.99), respectively.

**Conclusions:**

The extracts from *Datura Metel* L. showed the potential to treat psoriasis, and intravenous therapy might be a promising treatment to cure psoriasis, which is likely affected by selection and publication bias, still need more high quality clinical studies.

**Supplementary Information:**

The online version contains supplementary material available at 10.1186/s12906-023-04159-6.

## Introduction

Psoriasis is one of the most frequent chronic inflammatory skin diseases with cutaneous and systemic manifestations and considerable effects on patient quality of life [1]. It is estimated that psoriasis affects about 2–5% of the population in western countries, and approximately 1.42% in China[2–5]. Although there are multiple treatment for psoriasis mostly based on combating acute symptoms, there is still no cure for it [6–8].

*Datura metel* L. is the common species of the genus *Datura*. The dry flower of *Datura metel* L. is a traditional Chinese medicine, named Yangjinhua, which was first recorded in the Official compilation of *Taiping ShengHui Fang* (992 A.D.). Especially, *Yixue Zhongzhong Canxi Lu* (1909 A.D.) mentioned the effect of *Datura metel* L. to treat skin disease. In 1980s, the extracts from *Datura metel* L. were used to treat psoriasis in hospital [9–14]. At present, compounds isolated and identified from *Datura metel* L. mainly include alkaloids, withanolides, flavonoids and lignans, among which, alkaloids are one of the most important compounds in *Datura metel* L., with the maximum content up to 0.43%, and the representative compounds are atropine and scopolamine [15]. In *Chinese Pharmacopoeia* (2020 edition), *Datura metel* L. is available for treatment of skin diseases, and its main active component scopolamine is no less than 0.15% [16]. Rational administration of *Datura metel* L. is permitted in China.

In recent years, some experimental studies have found that the active components of *Datura metel* L. may treat psoriasis by regulating the balance of Treg/Th17 axis and inhibiting inflammatory cytokines production [17, 18]. However, the mechanism of the *Datura metel* L. therapy to treat psoriasis remains unclear, and further widespread evidence-based basic and clinical studies are needed to confirm its safety and efficacy.

In this study, we systematically review the literature and analyze the pooled effectiveness of the extracts from *Datura metel* L. in psoriasis patients using meta-analysis. We also assessed the safety and recurrence of treatment with the extracts from *Datura metel* L. in psoriasis patients. The findings of this study might provide insights into clinical treatment for psoriasis.

## Methods

### Outcome measures

The primary outcome measures were the cure rate in psoriasis patients, (1) the skin lesions completely or nearly completely disappear ( > = 90% of reduction in psoriasis area) and (2) subjective symptoms disappear. The secondary outcomes were the effective rate in psoriasis patients, with more than 30% skin lesions disappeared.

### Eligibility criteria

According to Population, Intervention, Comparator, Outcomes (PICO) criteria, our eligibility criteria were a patient population of psoriasis, and an intervention of extracts from *Datura metel* L. (intravenous injection or oral) with or without other interventions. Outcomes were the cure rate and effective rate defined by the researchers. In our research, study types included randomized controlled trials (RCT) and non-RCT, as prospective cohort studies, prospective registries, retrospective cohort studies, case series, and case reports.

### Information sources

We achieved English information from Pubmed (https://pubmed.ncbi.nlm.nih.gov), Embase (https://www.embase.com) and Cochrane Library (https://www.cochranelibrary.com). Chinese information sources were CNKI (https://www.cnki.net), Wanfang (https://www.wanfangdata.com.cn) and CQVIP (http://www.cqvip.com). All information dated from database inception until to September 2021. The primary term was “psoriasis”; secondary terms were: *Datura metel* L. or scopolamine or atropine. In addition, the reference lists from prior review articles were cross-referenced to identify additional articles.

### Data assessment and collection

2 independent reviews (X.Sang. and Y.T.) performed eligibility assessments in an unblended standardized manner. If there are disagreements between reviewers, and could not be resolved by consensus, the disagreement was resolved by a senior reviewer (F.W.). Both reviewers (X.Sang. and Y.T.) collected data by using a standardized data extraction sheet created specifically for this review, and the extracted data from each reviewer were then checked by the other reviewer. Unreported data points were termed NR (not reported). The data extracted from studies included: age, sex, country, classification, stage, course, treatment, cured cases, effective cases, the follow-up recurrence and adverse events.

### Quality assessment

Modified Jadad scale was used to assess the risk of bias in included RCT [19]. Methodological index for non-randomized studies (MINORS) were used to assess single-arm studies (non-randomized studies) [20]. The Joanna Briggs Institute (JBI) Critical Appraisal Checklist for case series were used to assess the retrospective studies [21].

### Data analysis

All data analyses were performed using Stata version 13.0 (StataCorp LP). We pooled the categorical variables as rates with 95% confidence intervals (CIs). A random-effect model was used under the assumption that data comes from varied populations with different distributions. The magnitude of heterogeneity was assessed by I^2^ statistic. For studies with a more than 80% incidence rate, the synthesized data were transferred using Freeman-Tukey double arcsine to avoid the anomalous values of 95%CI. Meta-analyses, heterogeneity testing, and bias risk assessment were undertaken in consultation with a statistician.

## Results

### Study selection and characteristics

**Fig. 1:**
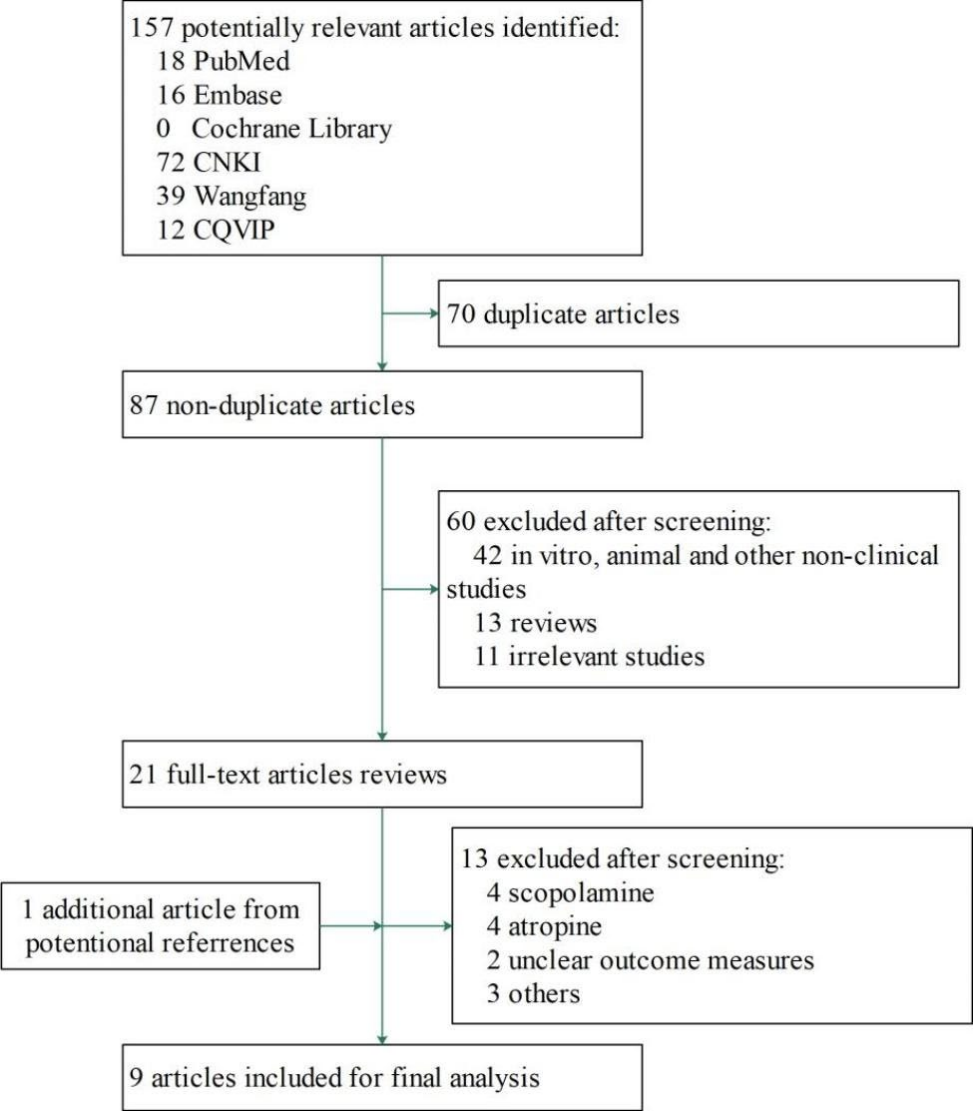
Flow chart of case series and single-arm study selection

The systematic search of Pubmed (n = 18), Embase (n = 16), Cochrane Library (n = 0), CNKI (n = 72), Wanfang (n = 39) and CQVIP (n = 12) yield 91 records. After duplicate removal, independent review of titles/abstracts by both reviewers (n = 87), full-text assessment (n = 21), cross-referencing from prior review reference lists (n = 1), and exclusion of ineligible full-text articles (n = 13), nine articles were included in the final review [9–14, 22–24]. The study selection process is shown in Fig. 1. Nine studies comprised 1778 patient were analyzed in this study. There were two different routes of administration, intravenous injection and oral. Five studies used intravenous therapy with 810 patients, and four studies used oral therapy with 968 patients. A summary of study characteristics is provided in Table 1. The details of extraction process of *Datura Metel* L. injection and capsule are in Supplementary Material and Table.

**Table 1:** Main characteristics of the studies included in the meta-analysis

Study ID	Location	Study type	Administration	Dosage	Total cases	Gender, male/female	Age	Classification				Stage		Course	Outcomes		Recurrence (follow-up)	adverse events
								PV	AP	EP	PP	Stabe	Unstable		Cured cases	Effective cases		
Liu et al. (1980)	China	Retrospective study	Intravenous	0.3 mg/kg, once	18	7/11	27.5 ± 9.3	NR	NR	NR	NR	2	16	11.4 ± 7.0(3 months-28 years)	5	15	3(NR)	NR
Liu et al. (1983)	China	Retrospective study	Intravenous	0.1 mg/kg, once	250	152/98	9–50	230	5	7	8	135	115	2 months-25 years	85	248	45(6 months)	0
Wang et al. (1985)	China	Single-arm study	Intravenous	0.4 mg/kg, once; 0.2 mg/kg, once; 0.15 mg/kg, once	242	129/113	10–69	238	1	1	2	66	176	1 month-35 years	163	210	6(6 months) 8(1 year) 19(2.5 years)	NR
Zhou et al. (1986)	China	Retrospective study	Intravenous	0.2 mg/kg, once	100	60/40	9–55	99	0	0	1	74	26	20 days-28 years	56	85	2(6 months)	NR
Qing et al. (1989)	China	Retrospective study	Intravenous	2ml, once every two week	200	NR	NR	200	0	0	0	NR	NR	NR	95	187	NR	NR
Chen et al. (1989)	China	Retrospective study	Oral + Extra-apply	unclear dose; once	670	NR	NR	NR	NR	NR	NR	NR	NR	1 month-40 years	203	589	NR	74.57%
Kang et al. (1999)	China	Single-arm study	Oral + Extra-apply	2 g, twice in a course of treatment	210	142/68	13–58	NR	NR	NR	NR	NR	NR	several months:25, 2–3 years:140, >4 years: 45	147	202	8(4 years)	NR
Zhou et al. (2011)	China	non-RCT study	Oral	3.3 g, three times in a course of treatment	58	36/22	14–53	58	0	0	0	NR	NR	3 months- 26 years	34	57	NR	4
Yang et al. (2018)	China	RCT study	Oral + Extra-apply	100 mg, bid	30	17/13	34.6 ± 3.5	30	0	0	0	NR	NR	6.6 ± 1.5 months	3	28	NR	NR

PV, Psoriasis vulgaris. AP, Arthropathic psoriasis. EP, Erythrodermic psoriasis. PP, Pustular psoriasis

### Quality assessment

There are one RCT study, one non-RCT study, two single-arm studies and five retrospective studies. One RCT study [22] was low-quality that scored 2 points through modifies Jadad scale. One non-RCT study [23] and two single-arm studies [12, 24] were assessed using the MINORS index scored from 7 to 9, which were acceptable for the present meta-analysis. Five retrospective studies [9–11, 13, 14] without comparison were assessed using the JBI Critical Appraisal Checklist for Case Series. Their overall appraisal were “include”. The details of the assessment are provided in Table 2.

**Table 2:** Quality assessment of included studies

Retrospective study											
Study ID	Q1	Q2	Q3	Q4	Q5	Q6	Q7	Q8	Q9	Q10	Overall appraisal
Liu et al. (1980)	Yes	Yes	Unclear	No	No	Yes	Unclear	Yes	No	Yes	Include
Liu et al. (1983)	Yes	Yes	Unclear	Yes	No	Yes	Yes	Yes	No	Yes	Include
Zhou et al. (1986)	Yes	Yes	Unclear	Yes	No	Yes	Yes	Yes	No	Yes	Include
Chen et al. (1989)	Yes	Yes	Unclear	No	No	Yes	Unclear	Yes	No	Yes	Include
Qing et al. (1989)	Unclear	Unclear	Unclear	No	No	No	No	Yes	No	Yes	Include
**Single-arm & non-RCT study**											
**Study ID**	**I**	**II**	**III**	**IV**	**V**	**VI**	**VII**	**VIII**			**Total**
Wang et al. (1985)	2	2	1	2	0	2	0	0			9
Kang et al. (1999)	2	2	0	2	0	2	0	0			8
Zhou et al. (2011)	2	2	1	2	0	0	0	0			7
**RCT study**											
**Study ID**	**Generation of allocation sequence**		**Allocation concealment**		**Investigator blindness**		**Withdrawals and dropouts**		**Efficacy of randomization**		**Total**
Yang et al. (2018)	1		1		0		0		0		2

Q1, Were there clear criteria for inclusion in the case series? Q2, Was the condition measured in a standard, reliable way for all participants included in the case series? Q3, Were valid methods used for identification of the condition for all participants included in the case series? Q4, Did the case series have consecutive inclusion of participants? Q5, Did the case series have complete inclusion of participants? Q6, Was there clear reporting of the demographics of the participants in the study? Q7, Was there clear reporting of clinical information of the participants? Q8, Were the outcomes or follow-up results of cases clearly reported? Q9, Was there clear reporting of the presenting site(s)/clinic(s) demographic information? Q10, Was statistical analysis appropriate?

I, a clearly stated aim; II, inclusion of consecutive patients; III, prospective collection of data; IV, endpoints appropriate to the aim of the study; V, unbiased assessment of the study endpoint; VI, follow-up period appropriate to the aim of the study; VII, loss of follow up less than 5%; VIII, prospective calculation of the study size. The items are scored 0 (not reported), 1 (reported but inadequate) or 2 (reported and adequate)

### Primary and secondary outcomes

The curative effect evaluation of patients with psoriasis is usually based on the recovery of lesions. Most of the included studies reported a cure for psoriasis with *Datura Metel* L. therapy. According to the criteria of curative effect described in these articles, the primary outcome measures were the cure rate in psoriasis patients, (1) the skin lesions completely or nearly completely disappear ( > = 90% of reduction in psoriasis area) and (2) subjective symptoms disappear. The secondary outcomes were the effective rate in psoriasis patients, with more than 30% skin lesions disappeared.

**Fig. 2:**
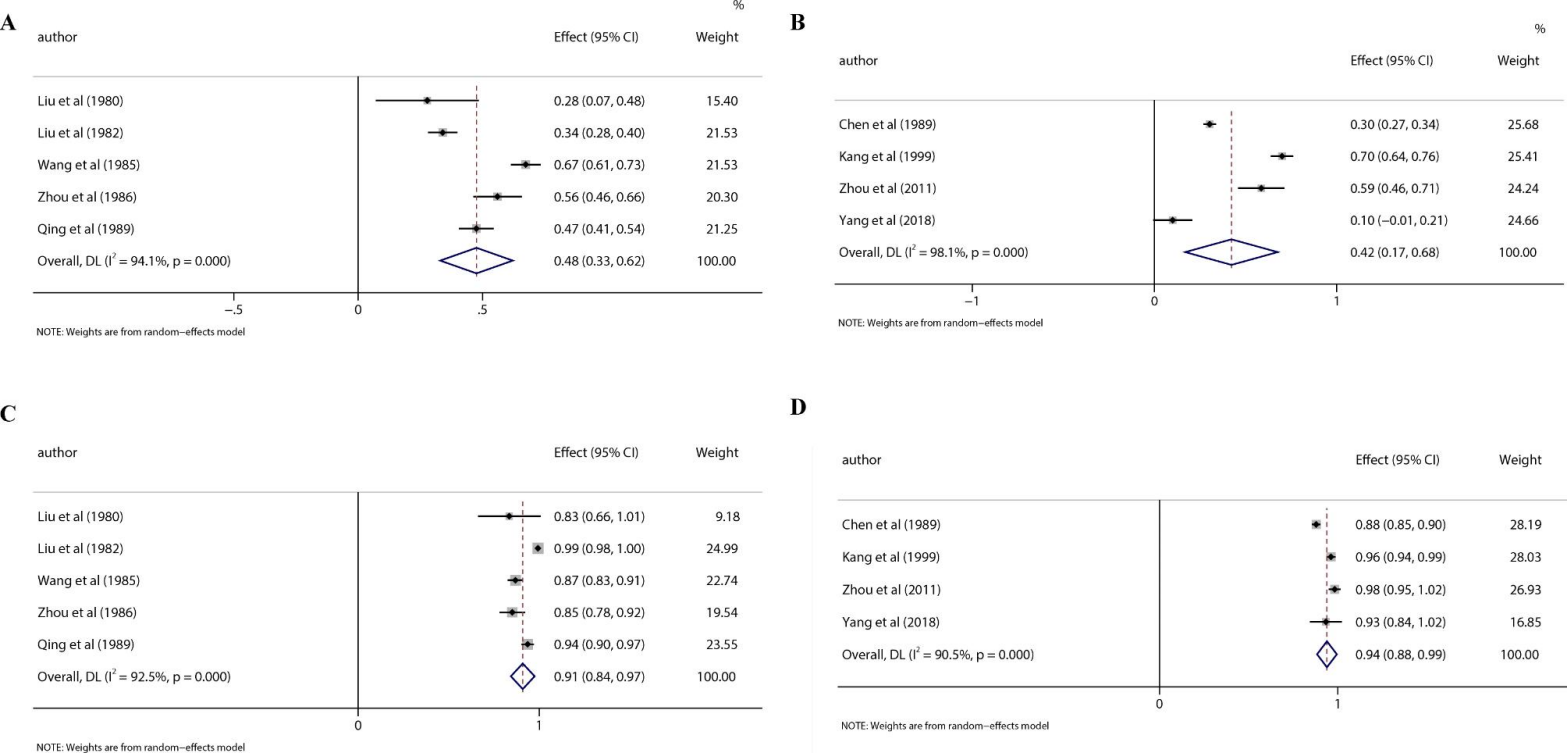
Forest plot. **A**, Cure rate of *Datura Metel* L. intravenous therapy. **B**, Cure rate of *Datura Metel* L. oral therapy. **C**, Effective rate of *Datura Metel* L. intravenous therapy. **D**, Effective rate of *Datura Metel* L. oral therapy

Primary and secondary outcome results were analyzed by two different routes of administration, and were reported in Fig. 2. The case cure rate of *Datura Metel* L. intravenous therapy was 0.48 (95% CI: 0.33, 0.62. I^2^: 94.1%) and of *Datura Metel* L. oral therapy was 0.42 (95% CI: 0.17, 0.68. I^2^: 98.1%), respectively. The case effective rate of *Datura Metel* L. intravenous therapy was 0.91 (95% CI: 0.84, 0.97. I^2^: 92.5%) and of *Datura Metel* L. oral therapy was 0.94 (95% CI: 0.88, 0.99. I^2^: 90.5%), respectively.

### Safety and recurrence

Three studies reported the number of adverse events, 74.57% [9], 6.9% [23]and 0% [13], respectively. Those adverse effects from general anesthesia include dilated pupils, blurred vision, and even restlessness, which can be treated. Other studies have noted that some patients experience increases in heart rate, blood pressure and body temperature during treatment, but recover naturally within a few hours [11, 12].

Five studies had followed patients after treatment [11–14, 24]. The recurrence rates at 6 months were between 2 and 18% [11–13].

## Discussion

Psoriasis is a disorder of both the innate and the adaptive immune systems, which lead to sustained inflammation [8]. Typical clinical manifestations of skin lesions are scaly erythema or plaques, localized or widely distributed. As far as now, advances in the treatment of psoriasis have been limited, barely no case report can cure psoriasis and finding a cure for psoriasis is urgent [1, 8].

*Datura metel* L., as a traditional Chinese medicine, has been used to anesthetize patients for at least one thousand years. Since 1970, the alkaloids in *Datura metel* L. have been made into injections and used widely as general anesthetics. During 1980 to 1988, *Datura metel* L. injection was applied to treat psoriasis and achieved great progress. In the late 1988, *Datura metel* L. was limited as toxic drug by the government and no longer used as before. Laterly, with the further standardization of *Chinese Pharmacopoeia*, usage of *Datura metel* L. restarted again gradually and more and more studies on its components have been conducted [15, 16]. So, it’s important to review the previous studies.

Interestingly, we find the articles which record the methods and results in treating psoriasis by *Datura metel* L. injection, and the range in cure rate from 28 to 67% [10–14]. Intravenous therapy for psoriasis is using a mixture of analgesic and phenothiazine derivatives as a premedication in advance before applying *Datura metel* L. injection in anesthesia. Patients can sleep deeply for 6 to 8 h and wake up naturally [11–14]. Oral therapy for psoriasis is taking *Datura metel* L. capsules with or without using diazepam to put the patient to sleep half hour later [9, 11, 24]. In this meta-analysis, the case cure rate in intravenous therapy is higher than that in oral therapy, and the effective rate in both are closely (Figs. 1 and 2). This difference may be related to the different extraction process of *Datura Metel* L. injection and capsule. The content of total alkaloids or withanolides in *Datura metel* L. injection was higher than capsule.

In those cured cases, most patients stopped itching as soon as they woke up from anesthesia, and had significant desquamation in 3 to 7 days, the skin lesions completely or nearly completely disappear in 1 to 3 months [11–14]. Adverse effects from *Datura metel* L. therapy could be treated or avoided. According to the considerable cure or effective rate, *Datura metel* L. therapy might be a promising treatment for patients suffering from psoriasis, particularly for the severe or recalcitrant types.

Given the period in which these studies were conducted, the quality of clinical studies was not high (Table 2). However, the case reports described complete diagnosis and treatment in details, which can give confidence in the results [13, 14]. As the psoriasis area and severity index (PASI) wasn’t been proposed at that time, the researchers used their own agreed-upon criteria to determine efficacy, resulting in high heterogeneity (Figs. 1 and 2). Hence, large sample size and control groups with unified criteria should be executed in further studies.

In addition, further research to clarify the underlying basic mechanisms of *Datura metel* L. therapy for psoriasis is warranted. Natural products extracted from herbal medicines have structural diversity and multiple active mechanisms, which have been proved to have synergistic effects to alleviate psoriasis and its comorbidities, including *Datura metel* L. [25]. Many effective components in *Datura metel* L. have been found, with a wide range of biological activities including antispasmodic, analgesia, anti-inflammatory, immunosuppressive, anti-allergic, and other pharmacological effects [26]. Total withanolides from *Datura metel* L. have been clarified that the improvement of the imbalance of the Treg/Th17 axis may be the key immunological mechanism in the treatment of psoriasis in the IMQ-induced mouse psoriasis model. Scopolamine and atropine are the main components of the total alkaloids in *Datura metel* L., inhibit the cerebral cortex and some parts under the cortex, accelerate the heart rate, dilate blood vessels, and improve the brain microcirculation [27]. The withanolides in *Datura metel* L. regulate both angiogenesis and inflammation, including sphingolipid metabolism and HIF-1-α/VEGF pathway [28]. These functions might improve the microcirculation of skin lesions and regulate the immune system through the nervous systems (in hypothesis). These researches studied the possible effects of *Datura metel* L. in the treatment of psoriasis, but the mechanism was not clarified enough. Moreover, it is important to find effective components of *Datura metel* L., reduce toxicity and increase efficiency for clinical application. This is probably the most promising drug for psoriasis. Still, more research is needed to understand the mechanism of *Datura metel* L. therapy.

This study had some limitations. Firstly, high heterogeneity existed in outcomes, and lots of factors could lead to heterogeneity, such as differences among various therapy regimens, disease type, disease stage, age, and evaluation criterion. Second, as single-arm trials lacked control groups, the comparison between other treatments was based on data from the population with a discrepant baseline.

## Conclusion

In conclusion, this meta-analysis suggested that *Datura metel* L. therapy could relieved or disappeared the skin lesions and itch of patients with psoriasis. The results of this study might support the extracts of *Datura metel* L. as a relatively considerable treatment option for patients with moderate-to-severe psoriasis. Large sample size and control groups are expected to confirm the efficacy and safety of *Datura metel* L. therapy in further studies.

### Electronic supplementary material

Below is the link to the electronic supplementary material.

**Supplementary Material 1**

## Data Availability

The data presented in the study are available from the corresponding author upon reasonable request.
