# Supplementary material for: Efficacy of extracts from Datura Metel L. for Psoriasis: a meta-analysis of case series and single-arm studies
Source: BMC Complement Med Ther. 2023 Sep 14;23:320. doi: 10.1186/s12906-023-04159-6 (PMC10500872; doi:10.1186/s12906-023-04159-6)
Supplement: Supplementary file 1 — Supplementary Material 1 [file 12906_2023_4159_MOESM1_ESM.docx]

**Efficacy of Extracts from** ***Datura Metel* L. for** **Psoriasis:** **A meta-analysis of case series and single arm studies**

**Supplementary material**

Xiaopu Sang^1^, Huanzhou Bi^1^, Xinlei Si^1^, Yihang Wang^1^, Xianjie Shi^1^, Fenfang Wu^1 *^

^1^Longgang Key Laboratory of Chinese Medicine and Immunology, Shenzhen Hospital, Beijing University of Chinese Medicine. Shenzhen, Guangdong, 518100, China, People’s Republic of China

^*^Corresponding author.

Corresponding author at: Longgang Key Laboratory of Chinese Medicine and Immunology, Shenzhen Hospital, Beijing University of Chinese Medicine. Shenzhen, Guangdong, 518100, China, People’s Republic of China.

E-mail address: wufenfang19@126.com

**The preparation process of *Datura Metel* L. injection**

The crude Yang Jin Hua (*Datura Metel* L.) powder was placed in a covered stainless steel bucket, stirred with 0.8 times of 45% ethanol solution, placed for 15 min, evenly loaded into the percolation cylinder, and slowly added 45% ethanol solution. After discharging the air between the powder particles and collecting 5 L of ethanol effluent, closing the discharge valve, covering the percolation cylinder and maceration.

After 24 h of maceration, the percolating solution was carried out and obtained with 20 times the amount of 45% ethanol and 1 mL/min flow rate. The ethanol solution and percolation solution were combined, ethanol was recovered, left to stand, and filtered to obtain filtrate 1. Three times 95% ethanol was added to filtrate 1, refrigerated at 4°C for 6 h. The upper clear layer was taken and the ethanol was recovered to obtain the concentrated solution. Petroleum ether was added to the concentrated solution (concentrated solution: petroleum ether = 1:2), extracted twice, and the petroleum ether was discarded to obtain the aqueous phase solution. The aqueous phase solution was adjusted to pH 10-11 with NaOH, and two times of chloroform was added to extract four times to obtain chloroform extraction solution 1. Add 1 times 2% hydrochloric acid to chloroform extraction solution 1, and then add 1 times 1% hydrochloric acid to extract 2 times, and combine the acid solution to obtain acid solution 1. The acid solution 1 was adjusted to pH 10-11 with NaOH and extracted 3 times with 1 times chloroform to obtain chloroform extract 2. Chloroform extraction solution 2 was extracted once by adding 1 times of 1% hydrochloric acid, and then 2 times extracted by adding 1 times of 0.5% hydrochloric acid, and the acid solution was combined to obtain acid solution 2, which was adjusted to pH 2-3, heated to recover chloroform, 1% activated carbon was added for decolorization, and filtered to obtain filtrate 2. To measure the amount of scopolamine in the filtrate, physiological saline was added until the content of scopolamine was 2.5 mg/mL, PH was adjusted to 5-6, 0.22 m ultrafiltration, canning, melt sealing, sterilization, quality testing, packaging, which was the *Datura Metel* L. injection (5mg/2mL, calculated as scopolamine content).

**The preparation process of *Datura Metel* L.** **capsule**

The powdered Yangjinhua (*Datura Metel* L.) is sieved through 100 mesh, and 0.25g of Yangjinhua (*Datura Metel* L.) powder is packed into capsules.

Supplementary table. The details of *Datura Metel L.* injection and capsule.

| **Botanical or multiherbal** | | | | |
| --- | --- | --- | --- | --- |
| **Study** | **Species, source, concentration** | **Quality control reported? (Y/N)** | **Chemical analysis reported? (Y/N)** | |
| Chen et al (1989) | *Datura metel L.,*250mg; wintermin 25mg; promethazine 25mg | N | N | |
| Kang et al (1999) | *Datura metel L.,*250mg; wintermin 25mg; promethazine 25mg | N | N | |
| Zhou et al (2011) | *Datura metel L.,*250mg; wintermin 25mg; promethazine 25mg | N | N | |
| **Patented formulations, botanical or chemical** | | | | |
| **Study** | **Formulation** | **Source** | **Quality control reported? (Y/N)** | **Chemical analysis reported? (Y/N)** |
| Yang et al (2018) | Yangjinhua Capsule | Prepared by Yang et al (2018) | Y | Y-HPLC |
| **Isolated chemical compound** | | | | |
| **Study** | **Compound, concentration** | **Source** | **Purity(%) (and grade, if applieable)** | **Quality control reported? (Y/N)** |
| Liu et al (1980) | Alkaloid from flower of *Datura metel* L., 0.3mg/kg* | Hospital preparations | 85% scopolamine, 15% hyoscyamine and atropine | N |
| Liu et al (1983) | Alkaloid from flower of *Datura metel* L., 0.1mg/kg | Hospital preparations | 85% scopolamine, 15% hyoscyamine and atropine | N |
| Wang et al (1985) | Alkaloid from flower of *Datura metel* L., 0.4mg/kg; 0.2mg/kg; 0.15mg/kg | Hospital preparations | 85% scopolamine, 15% hyoscyamine and atropine | N |
| Zhou et al (1986) | Alkaloid from flower of *Datura metel* L., 0.2mg/kg | Hospital preparations | 85% scopolamine, 15% hyoscyamine and atropine | N |
| Qing et al (1989) | Alkaloid from flower of *Datura metel* L., 5mg/2ml | Hospital preparations | unclear | N |

* 0.3mg per kilogram of body weight
